# Morphological abnormalities of leukocytes in SARS-CoV-2 infection

**DOI:** 10.4314/ahs.v23i4.13

**Published:** 2023-12

**Authors:** Moueden Amine, Messaoudi Reda, Derouiche Mokhtar

**Affiliations:** Université d'Oran 1 Ahmed Ben Bella Faculté de Médecine, Pharmacie

**Keywords:** COVID-19, peripheral blood smear, abnormalities, neutrophil, lymphocyte, monocyte

## Abstract

**Introduction:**

The causative agent of COVID-19 (Coronavirus Disease 2019) is an enveloped RNA (ribonucleic acid) virus of the SARS-CoV-2 (Severe Acute Respiratory Syndrome Coronavirus-2) family. The effects of SARS-CoV-2 on the differentiation and maturation of blood cells have been the subject of several studies, we report our experience of an investigation of the morphologic abnormalities of leukocytes observed during COVID-19.

**Patients and methods:**

This is a prospective study of 5 months, from February 2021 to June 2021. Forty COVID-19 patients and 20 healthy controls were included in this study. We performed complete blood count and peripheral blood smear of all patients and control samples. Leukocytes abnormalities were quantified as a percentage of 100 leukocytes of the same lineage.

**Results:**

The morphological abnormalities of the leukocytes found in percentage of patients have been mainly neutrophils bilobed 72,5%, hypogranulation 45%, acquired pseudo Pelger-Huet 35%, vacuolated neutrophils 42,5%, Apoptotic neutrophils 35,5 %, neutrophils with toxic granulations 30%, myelemia 45%, atypical lymphocytes 52,5%, lymphoplasmocytes 60% and vacuolated monocytes 27, 5%.

**Conclusion:**

Our study revealed several morphological abnormalities of the different cells of the leukocyte lineage. The presence of toxic granulations in the cytoplasm of the myelocytes was specific to this study.

## Introduction

The causative agent of COVID-19 (Coronavirus Disease 2019) is an enveloped RNA (ribonucleic acid) virus of the SARS-CoV-2 (Severe Acute Respiratory Syndrome Coronavirus-2) 1. Our understanding of COVID-19 is growing every day. It is a systemic disease that could disrupt the function of multiple organs 2.

In the peripheral blood, apart from the hypercoagulability observed in COVID-19, the virus causes quantitative and qualitative abnormalities of the various blood cells. It could be responsible for anaemia, neutrophil polynucleosis, lymphopenia, thrombocytopenia, as well as other disturbances of the quantitative data of the blood count 3, 4 . The effects of SARS-CoV-2 on the differentiation and maturation of blood cells have been the subject of several studies.

In this article we describe the morphological abnormalities of leukocytes observed in our series of COVID-19 patients.

## Patients and methods

This is a 5-month prospective study, from February 2021 to June 2021, which included 40 patients followed for complications related to COVID-19 at the COVID-19 units of the Oran University Hospital in Algeria. SARS-CoV-2 infection was confirmed in all cases by RT-PCR (Reverse Transcription-Polymerase Chain Reaction) analysis of nasopharyngeal swab specimens.

Blood samples were obtained on the day of patients' admission on an EDTA tube (Ethylene Diamine Tetra Acetic) and the blood count was done on an auto hematology analyzer Mindray BC - 6800®. A peripheral blood smear (PBS) was then performed by spreading a drop of peripheral blood along glass slide and staining with MGG (May Granwald and Giemsa). Morphological abnormalities of the leukocytes were highlighted after reading the PBS under the light microscope (magnification*100). Twenty non-COVID-19 peripheral blood smears (negative antigen test) were examined morphologically to act as Control samples. Leukocyte's abnormalities were quantified in each patient. Neutrophil's abnormalities were quantified as a percentage of 100 neutrophils, lymphocyte's abnormalities were quantified as a percentage of 100 lymphocytes and monocytes' abnormalities were quantified as a percentage of 100 monocytes.

### Statistical analysis

A threshold of 0.05 % was considered significant for all the statistical tests performed. The qualitative variables were expressed as a percentage or number of cases n on the total N; and the quantitative variables were expressed as mean and standard deviation. The data was entered and analysed on a computer support using the IBM SPSS statistics 21software.The comparison of the qualitative variables was made by the Fisher's exact test.

## Results

In our study, we report that 60% of the patients, were male and 40% were female (sex ratio M/F = 1.5). The average age was (58.75 ± 13.86 years). Forty percent of patients had anemia (78% normocytic, 16% microcytic, and 6% normocytic).

**Figure 1:**
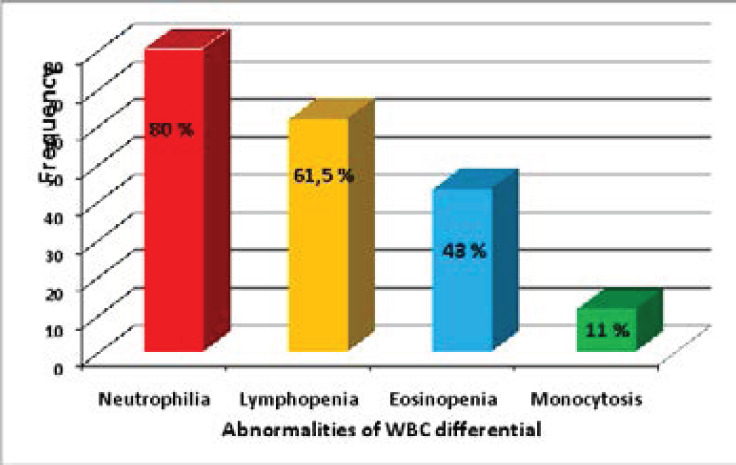
Frequency of quantitative leukocytes abnormalities in our COVID-19 cohort

**Figure 2:**
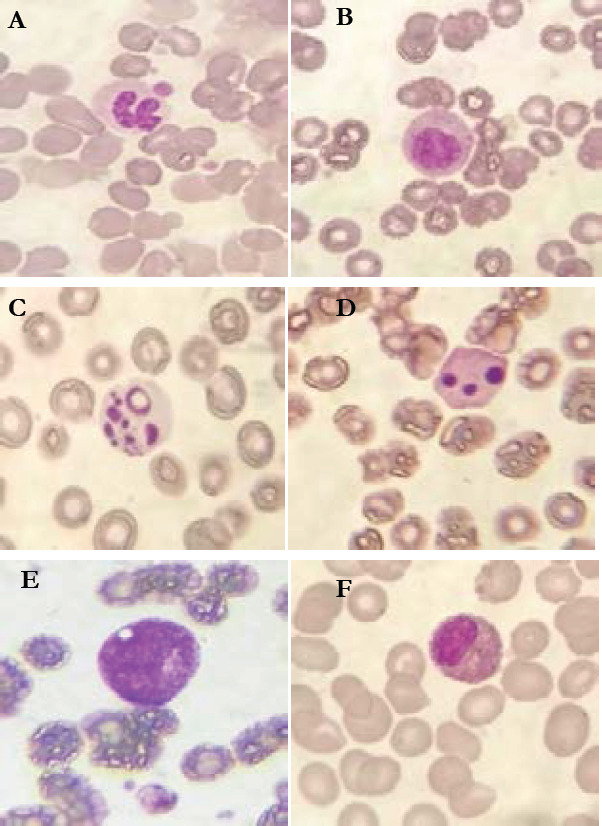
Morphological abnormalities in myeloid cells of COVID-19 patients (MGG * 100). A: Acquired Pelger-Huët abnormality. B C and D: Neutrophil in apoptosis. E: Hyper-granular vaculolated myelocyte. F: Single-lobed eosinophil

**Figure 3:**
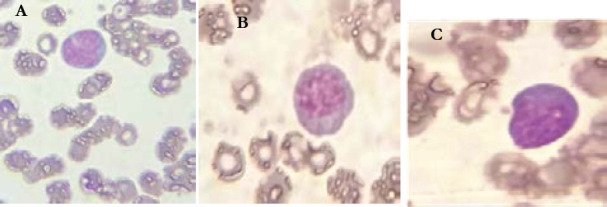
Morphological abnormalities of lymphocytes from COVID-19 patients (MGG * 100). A : Stimulated lymphocytes (Blaste like). B , C : lymphoplasmocyte

**Figure 4:**
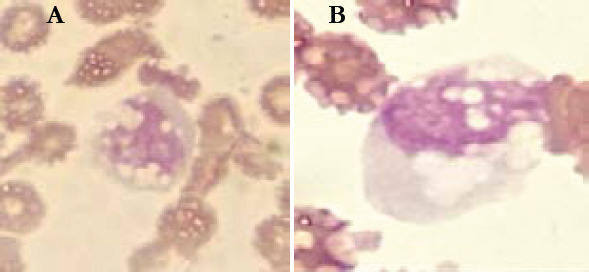
Morphological abnormalities of monocytes from COVID-19 patients (MGG * 100). A, B: hypervacuolated monocytes with giant vacuoles

Sixty seven percent of patients had hyperleukocytosis, while 10.6% of patients presented with leucopenia. Abnormalities of CBC differential counts in percentage of patients are summarized in figure 1. The morphological abnormalities of granulocytic lineage, lymphocytes and monocytes found in PBS are illustrated in the following figures (figure 2, figure 3, and figure 4).

The comparison between patients and controls for the frequency of morphological abnormalities show a significant difference for all abnormalities except atypical lymphocytes and vacuolated monocytes (Table 1), but monocytes with numerous and giant vacuoles are absent in control samples.

**Table 1:** Comparison between patients and controls for the frequency of morphological abnormalities

Abnormalities	Covid-19 group (N = 40) n /% of patients	Control group (N =20) N % of control samples	P	Average in % of 100 leukocytes of the same lineage ± standard deviationin patients Covid-19
Neutrophils bilobed	29 / 72,5%	1 / 5%	0.000	15,47 ± 15,59
Pseudo-pelger huet	14 / 35%	1 / 5%	0.012	0,92 ± 1,43
Hypogranulation	18 / 45%	3 / 15%	0,025	16,32 ± 23,62
Vacuolated neutrophils	13 / 32,5%	1 / 5%	0.011	9,27 ± 20,85
Neutrophils and myelocytes with toxic granulations	12 / 30%	0 / 0%	0.005	7,17 ± 14,99
Apoptotic neutrophils	15 / 37,5%	0 / 0%	0,001	0,72 ± 1,03
Myelemia	18 / 45%	1 / 5%	0.000	1,67 ± 2,48
Atypical lymphocytes	21 / 52,5%	5 / 25%	0.056	18,50± 22,07
Lymphoplasmocytes	24 / 60%	1 / 5%	0.000	4,30± 4,97
Vacuolated monocytes	11 / 27,5%	3 / 15%	0.34	0,55 ± 1,10

## Discussion

In the present study, the findings observed in WBC (White Blood Cell) differential were neutrophilia (80%). lymphopenia (61, 5%), monocytopenia (11%) and eosinopenia (16%). These corroborated with findings reported by several studies and have emerged as a hallmark of severe COVID-19. 5-8.

The abnormalities of leukocytes were found in 100 % of patients. If we compare the presence of these abnormalities in covid 19 patients and control samples, we find a significant difference between these two groups except atypical lymphocytes and vacuolated monocytes. The frequent sign of abnormalities of myeloid cells is hyposegmentation of the nucleus [neutrophils bilobed: 72 ,5 % of patients and (15,47 ± 15,59) of 100 leukocytes of all patients; pseudo Pelger-Huet: 35% of patients, (0,92 ± 1,43) of 100 leukocytes off all patients]. And 47.5 % of patients have a bilobed PNN count greater than 10 per cent.

In study of Nazarullah et al ; these 2 forms, have almost completely disappeared in all cases after one week of treatment with antiviral and anti-inflammatory drugs in study of Nazarullah et al 9. Apoptotic forms of neutrophils was also noted in 35, 5% of patients. All patients have a rate of apoptotic neutrophils lower than 5 % of neutrophils .These abnormalities have also been found in other studies but rarely 9, this is due to the action of inflammatory cytokines which delay apoptosis of neutrophils 10.

We also found myelemia in 45% of patients. Peripheral blood film examination of one patient revealed toxic granulations with vacuoles in their neutrophilic myelocytes. According to our literature search; this phenomenon was not present in any previous study. However, in the study by Singh et al, cytotoxic granulations were found in the cytoplasm of neutrophils but not in immature cell like myelocyte 11 . Hypogranulation of granulocytic cell was also noted in 45% of patients (7 patients have a hypogranulated neutrophil count greater than 50% of total neutrophils). This abnormality was also observed in other studies 12.

The results showed that atypical lymphocytes were found in approximately half of the COVID-19 patients. These were mainly the presence of atypical lymphocytes, stimulated lymphocytes, lymphoplasmocytes and plasma cells, thus making COVID-19 a new etiology of atypical lymphocytes 13, 14.

We note the presence of the blast-like cell; these are stimulated lymphocytes with diameter between 10 and 12 micrometer. These are a polymorphic nucleus with intermediate to fine chromatin. The cytoplasm is bleu without granules. Atypical lymphocytes may be present in healthy person, but in a very small percentage. They are mostly present in response to viral, bacterial or parasitic infections. 15, such as infectious mononucleosis 16. Nazrullah et al. found atypical lymphocytes in all COVID-19 positive patients 9 .

Lymphoplasmocytes are present in 60% of patients, of which 20.8% of patients have more than 10% of lymphoplasmocytes of all lymphocytes. Lymphoplasmocytes are also present in other similar studies with high frequencies 9, 17, 18. This phenomenon could be seen in certain inflammatory and infectious conditions, although in the study of Sadigh et al, the presence of plasma cells was particularly common in COVID-19 19.

We also found the presence of numerous giant vacuoles in the cytoplasm of monocytes; this is related to the main function of monocytes which is phagocytosis. These morphologic changes have been reported in other studies^20^. These findings are not very specific but monocytes with numerous and giant vacuoles are absent in control samples. Some authors assume that monocyte activation could be a good prognostic sign 21.

## Conclusion

Our study revealed several morphological abnormalities of the different cells of the leukocyte lineage. These abnormalities have also been described in other studies. However, the presence of toxic granulations in the cytoplasm of the myelocytes was specific to our study. Further studies with extensive sampling are needed to better investigate the relationship between the pathophysiology of COVID-19.
